# Targeting E2 ubiquitin-conjugating enzyme UbcH5c by small molecule inhibitor suppresses pancreatic cancer growth and metastasis

**DOI:** 10.1186/s12943-022-01538-4

**Published:** 2022-03-10

**Authors:** Simin Qi, Xiaoqing Guan, Jia Zhang, Dehua Yu, Xuefei Yu, Qinglin Li, Wenjuan Yin, Xiang-Dong Cheng, Weidong Zhang, Jiang-Jiang Qin

**Affiliations:** 1 The Cancer Hospital of the University of Chinese Academy of Sciences (Zhejiang Cancer Hospital), Institute of Basic Medicine and Cancer (IBMC), Chinese Academy of Sciences, Hangzhou, 310022 Zhejiang China; 2 College of Pharmaceutical Sciences, Zhejiang Chinese Medical University, Hangzhou, 310053 China; 3 Shanxi Institute of Traditional Chinese Medicine, Taiyuan, 030012 China; 4 Institute of Interdisciplinary Integrative Medicine Research, Shanghai University of Traditional Chinese Medicine, Shanghai, 201203 China; 5 School of Pharmacy, Second Military Medical University, Shanghai, 200433 China

**Keywords:** IAP, UbcH5c, Pancreatic cancer, Small-molecule inhibitor, NF-κB

## Abstract

**Background:**

Pancreatic cancer is one of the most lethal cancers worldwide. The IAPs function as E3 ubiquitin ligases and contribute to pancreatic cancer initiation, progression, and metastasis. Although IAP-targeted therapies have been developed and shown anticancer efficacy in preclinical settings, none of them has been approved yet.

**Methods:**

Transcriptome data from public datasets were used to analyze the correlation of IAPs and E2s, and the biological function of E2 UbcH5c in pancreatic cancer. A structure-based virtual screen was used to identify UbcH5c inhibitor, and surface plasmon resonance analysis and cellular thermal shift assays were employed to evaluate the binding affinity. The anticancer activities were demonstrated through in vitro and in vivo assays, while the related mechanisms were explored through transcriptomic and proteomic analyses and confirmed by western blot, immunofluorescence, and qRT-PCR.

**Results:**

UbcH5c is positively correlated with the expression of IAPs in pancreatic cancer. We further found that UbcH5c is overexpressed and associated with a poor prognosis in pancreatic cancer. We identified a small-molecule UbcH5c inhibitor, termed DHPO, which directly bound to UbcH5c protein. DHPO inhibited cell viability and colony formation, induced apoptosis, and suppressed migration and invasion of pancreatic cancer cells in vitro. The compound inhibited UbcH5c-mediated IκBα degradation and NF-κB activation, which is critical for its anticancer activity. Furthermore, DHPO suppressed the tumor growth and metastasis in two orthotopic pancreatic tumor mouse models.

**Conclusions:**

These results indicated that inhibiting UbcH5c is a novel and effective strategy for treating pancreatic cancer and DHPO represents a new class of UbcH5c inhibitor and may be further developed as an anti-pancreatic cancer therapeutic agent.

**Supplementary Information:**

The online version contains supplementary material available at 10.1186/s12943-022-01538-4.

## Background

Pancreatic cancer is a highly invasive malignant tumor worldwide characterized by late diagnosis and limited therapeutic options [1]. Based on tumor resectability, patients with pancreatic cancer have been classified into four categories: resectable, borderline resectable, locally advanced, and metastatic [2]. In spite of recent advances in the early diagnosis and treatment of pancreatic cancer, complete surgical resection is the only way to offer a chance of cure for patients with pancreatic cancer. Most cases (up to 80%) are diagnosed at advanced stages and lose the opportunity to carry out surgery. Chemotherapy combinations, mainly including gemcitabine plus nab-paclitaxel and FOLFIRINOX (made up of folinic acid, fluorouracil, irinotecan, and oxaliplatin) remain the primary treatment for patients with advanced disease [3, 4]. However, they have shown limited efficacy and severe adverse effects, and the combined five-year survival rate for pancreatic cancer is very low at just 5% to 10%. PARP inhibitors, such as olaparib, have gained approval for use in patients with germline *BRCA1* or *BRCA2* mutation [5]. Compared with placebo, olaparib improved median progression-free survival from 3.8 to 7.4 months, but there is no significant difference in overall survival between the groups. Thus, pancreatic cancer remains a deadly malignancy with limited options for effective therapy, and further development of more effective and better-tolerated treatments is warranted.

The ubiquitin–proteasome system provides opportunities for pharmacological intervention owing to their crucial roles in cancer [6]. A series of critical enzymes including ubiquitin-activating enzymes (E1), ubiquitin-conjugating enzymes (E2), ubiquitin-ligating enzymes (E3), and deubiquitinases are involved in the ubiquitin signaling, controlling post-translational ubiquitination of proteins and regulating protein stability [6]. To initiate protein modification by ubiquitin, an active site cysteine of E1 reacts with the C-terminus of ubiquitin. Then the ubiquitin is transferred from E1 to E2 followed by E3 directly or indirectly promoting the transfer of ubiquitin from E2 to the target substrate proteins. Removal ubiquitin from modified substrates is accomplished by deubiquitinases [7].

There are more than 600 known E3 ubiquitin ligases and they have been classified into three general types based on unique topology and mechanism: homologous to E6AP C-terminus (HECT), really interesting new gene (RING), and RING-between-RING (RBR) [8]. RING-type E3s represent the largest class of E3s in humans, are characterized by a conserved structure stabilized by two Zn^2+^ atoms, and catalyze the direct transfer of ubiquitin from E2-ubiquitin to a CO-bound substrate protein depending on the RING finger domain. Many of the RING-type E3s have been demonstrated as effective molecular targets for treating human cancers, including pancreatic cancer [9–11]. Among them, the inhibitor of apoptosis proteins (IAPs), including family members cIAP1/2 and XIAP, are implicated in cancer initiation, metastasis, and progression [12]. In pancreatic cancer, cIAP1 is over-amplified and high cIAP2 expression is correlated with resistance to chemotherapeutic drugs [12]. Co-expression of cIAP1/2 has been associated with an unfavorable prognosis of pancreatic cancer. Similar associations have been found between XIAP levels and pancreatic cancer prognosis [13, 14]. Accumulated evidence supports that IAPs play critical roles in numerous cellular processes such as induction of the epithelial-mesenchymal transition (EMT), DNA repair, and activation of NF-κB signaling, and have been demonstrated as promising targets for cancer therapy [12]. To date, medicinal chemistry programs directed at IAPs have resulted in clinically evaluated molecules, and only very few compounds targeting IAPs entered clinical trials and are still at the early phase [15].

Recent studies have demonstrated that targeting the E2-E3 axis is a potential strategy for developing anticancer agents [6]. The human proteome contains about 50 E2s and numerous studies have reported that E2s also serve an important role in the carcinogenesis and progression of pancreatic cancer [16]. UbcH10 expression at mRNA and protein levels are both upregulated in pancreatic ductal adenocarcinoma and is significantly correlated with poor overall survival [17]. UBE2C is upregulated in pancreatic cancer and associated with clinical stage, lymph node metastasis, and survival [18]. Silencing UBE2C inhibits cell proliferation by inducing cell cycle arrest and apoptosis and decreases the migration in vitro and in vivo. UBE2T has been recently identified as an oncogenic protein that is highly expressed in pancreatic cancer tissues and cell lines, and overexpression of UBE2T significantly promotes pancreatic cancer cell proliferation, migration, and invasion [19]. Increased UBE2S level promotes EMT via the VHL/HIF-1α/STAT3 signaling by attenuating the activity of the promoter [20]. UBE2N interacts with TRIM11 to decrease the sensitivity of Panc1 and BxPC3 cells to gemcitabine [21]. Collectively, targeting E2 enzymes may be an attractive strategy to modulate E3 ubiquitin ligases for pancreatic cancer therapy. Identifying E2s that collaborate with IAPs may also provide new molecular targets for developing effective anticancer agents.

In this study, we examined the expression of E2 UbcH5c in pancreatic cancer tissues and analyzed its association with IAPs expression and the prognosis of pancreatic cancer patients. We further identified a small-molecule UbcH5c inhibitor DHPO and investigated its anticancer activity and mechanisms of action in pancreatic cancer models in vitro and in vivo. Our data suggested that UbcH5c may be a promising target for anti-pancreatic cancer drug development.

## Methods

### Cell lines and chemicals

Cell lines Panc1, BxPC3, SW1990, HPAC, CFPAC1, AsPC1, Capan2, and HPNE were purchased from American Type Culture Collection (ATCC; Rockville, MD, USA). Panc1, BxPC3, SW1990, and HPNE were cultured in DMEM medium (Gibco/Life Technologies, Darmstadt, Germany) supplemented with 10% fetal bovine serum (FBS) (Thermo Fisher Scientific, Waltham, MA, USA). CFPAC1 and AsPC1 were cultured in RPMI 1640 medium (Gibco/Life Technologies, Darmstadt, Germany) supplemented with 10% FBS. Capan2 was cultured in IMDM medium (Gibco/Life Technologies, Darmstadt, Germany) supplemented with 20% FBS. HPAC was cultured in DMEM/F12 medium supplemented with 10% FBS and 0.002 mg/ml insulin, 0.005 mg/ml transferrin, 40 ng/ml hydrocortisone, and 10 ng/ml epidermal growth factor. All cell culture media were supplemented with 1% penicillin and streptomycin (Thermo Fisher Scientific/Life Technologies, Waltham, MA, USA) and incubated at 37 °C in a humidified atmosphere of 95% air and 5% CO_2_. The isolation and purification of DHPO were performed as previously [22]. TNF-α (Cat. No. Z01001) was purchased from GenScript. The anti-GAPDH (Cat. No. 5174S, 1/1000), anti-phospho-IκBα (Cat. No. 2859S, 1/1000), anti-IκBα (Cat. No. 4814S, 1/1000), anti-phospho-NF-κB p65 (Cat. No. 3033S, 1/1000), anti-NF-κB p65 (Cat. No. 8242S, 1/1000), anti-c-Myc (Cat. No. 18583S, 1/1000), anti-Mcl-1 (Cat. No. 94296S, 1/1000), and anti-UbcH5c (Cat. No. 4330S, 1/1000) antibodies were purchased from Cell Signaling Technology (Beverly, MA, USA).

### In vitro assays for determination of DHPO’s anticancer activity

All the assays used to evaluate the effects of DHPO on pancreatic cancer cell viability (CCK8 assays, Nuoyang Biotech, Hangzhou, China), colony formation, cell apoptosis (Annexin V-FITC Apoptosis Staining/Detection Kit, BD, USA), cell migration (wound healing assay), and cell invasion (transwell invasion assay) were performed as described previously [23, 24].

### Western blot

The methodology used here was described elsewhere [25]. In brief, the total protein was extracted from cells using RIPA buffer containing phosphatase and protease inhibitors (Sigma-Aldrich, St. Louis, MO, USA). The cell lysates were evaluated for their protein concentrations, resolved by SDS-PAGE, and transferred to polyvinylidene difluoride (PVDF) membrane (Millipore, Germany). The membranes were blocked in 5% skim milk and incubated with primary antibody followed by incubation with appropriate secondary antibody. ECL Chemiluminescent Substrate Reagent Kit (Biosharp, Hefei, China) was used to detect protein bands.

### Immunofluorescence

Confocal immunofluorescence was performed as described earlier [26]. In short, cells were seeded on glass coverslips overnight, treated with DHPO for 24 h followed by exposure to TNF-α (20 ng/ml) for 15 min. The treated cells were fixed and incubated with primary antibody followed by incubation with secondary antibody. Coverslips were then mounted with an anti-fade mounting solution supplemented with 4’,6-diamino-2-phenylindole (DAPI). The images were then captured using a confocal microscope (Leica Microsystems, Wetzlar, Germany).

### Real-Time qRT-PCR

The mRNA expression levels of genes were determined by Real-Time qRT-PCR as described previously [26]. Total RNA of cell samples was extracted using RNA-Quick Purification Kit (YiShan Biotechnology Co. LTD, Shanghai, China) according to the manufacturer’s instructions. The concentrations of isolated RNA samples were determined using NanoDrop (Thermo Fisher Scientific, Wilmington, DE, USA). The cDNA was synthesized using Fast-All-in-One RT Kit (YiShan Biotechnology Co. LTD, Shanghai, China) according to the manufacture’s protocols. Real-Time PCR was performed using 2xSuper SYBR Green Qpcr Master Mix (YiShan Biotechnology Co. LTD, Shanghai, China) and by means of CFX96™ PCR instruments (Bio-Rad Laboratories, Hercules, CA, USA). The data were analyzed using Bio-Rad CFX Manager™ Software version 3.1 (Bio-Rad Laboratories, Hercules, CA, USA). GAPDH was used as an internal control to normalize individual gene expression levels. Primer sequences were shown in Additional file 1: Table S1.

### Stable transfection

Lenti-shUbcH5c and their corresponding control vectors were constructed by and purchased from Hanbio (Shanghai, China). Stable transfection was performed according to the manufacturer's instructions. Then the stably transfected cells were screened by puromycin for 2 weeks.

### RNA sequencing and bioinformatic analysis

Panc1 cells were treated with 5 μM DHPO, or an equal volume of 1‰ DMSO as controls, and were incubated in a humidified atmosphere of 5% CO_2_ at 37℃ for 24 h. Cell samples were collected and added into Trizol reagent (Invitrogen, CA, USA), and then handed over to LC-Bio Technology Co., Ltd (Hangzhou, China) for the subsequent mRNA library construction and sequencing. Hisat2, StringTie, and Ballgown are used to compare the filtered CleanData with the human genome (ftp://ftp.ensembl.org/pub/release-101/fasta/homo_sapiens/dna/), and to perform initial assembly and FPKM quantification of genes or transcripts. All genes were used to perform PCA. PCA results were visualized in a two-dimensional coordinate space according to two major principal components. The differentially expressed mRNAs were selected with |log2FC|> 0.58 and *FDR* < 0.05 by R package edgeR. The genes with log2FC < -0.58 and *FDR* < 0.05 were selected to perform GO enrichment and KEGG enrichment analyses using DAVID. GSEA was performed with Broad GSEA software (version 4.0.2) using the hallmark gene sets (h.all.v.7.4.symbols.gmt) in MSigDB for pathway annotation.

### Quantitative proteome analysis

Panc1 cells were treated with 5 μM DHPO or DMSO for 24 h, and the cell samples were collected for proteome analysis with TMT technology by LC-Bio Technology Co., Ltd. Quantitative proteome analysis involved the following six steps in turn: protein extraction, SDS-PAGE separation, filter-aided sample preparation (FASP Digestion), TMT labeling, peptide fractionation with reversed-phase (RP) chromatography, and mass spectrometry analysis. Six samples were sequentially labeled with 126, 127, 128, 129, 130, and 131. MS/MS raw files were processed using the MASCOT engine (Matrix Science, London, UK; version 2.6) embedded into Proteome Discoverer 2.2. A peptide and protein false discovery rate of 1% was enforced using a reverse database search strategy. All proteins were used to perform PCA. PCA results were visualized in a two-dimensional coordinate space according to two major principal components. Proteins with |log2FC|> 0.26 and *FDR* (Student’s *t*-test followed by multiple test correction through the Benjamini–Hochberg method) < 0.05 were considered to be differentially expressed proteins. The top 150 downregulated proteins with *FDR* smaller than 0.05 were selected to perform pathway and process enrichment analysis, and protein–protein interaction enrichment was performed using Metascape. All these procedures were performed with the default configuration.

### Molecular docking

Molecular docking was carried out using Autodock 4.2 software. The crystal structure of UbcH5c (PDB code: 5EGG) was retrieved from the Research Collaboratory for Structural Bioinformatics Protein Data Bank (RCSB PDB, http://www.rcsb.org/pdb/). The standard structure of DHPO (PubChem CID: 286761) was retrieved from the PubChem Compound Database (https://pubchem.ncbi.nlm.nih.gov/). The Lamarckian genetic algorithm (LGA) was used to implement molecular docking.

### Surface plasmon resonance analysis

The binding affinity of DHPO to UbcH5c was evaluated using a Biacore 8 k system (GE Healthcare, Sweden) at 25 °C. After diluting with 50 mM acetic acid sodium acetate buffer, 20 μg/ml of UbcH5c protein was immobilized onto a CM5 sensor chip at a flow rate of 30 μl/min in PBS containing 5% (v/v) DMSO and 0.05% (v/v) Tween-20. Gradient concentrations of DHPO were injected into the flow system and analyzed, respectively. The UbcH5c protein and DHPO were allowed to associate for 120 s and then dissociate for 180 s. After dissociation, the sensor chip was washed with running buffer and regenerated with pure water containing 10% DMSO for 30 s. The injection mode of the sample is multi-cycle kinetic injection. The concentration of 0 is deducted in all the data to obtain the final binding dissociation curve.

### Cellular thermal shift assays

After treatment with DHPO or DMSO for 1 h, Panc1 and SW1990 cells were digested with trypsin, centrifuged, then resuspended in pre-cooled PBS supplemented with protease inhibitors, and transferred to the PCR tubes. PCR tubes were heated at their designated temperature for 3 min in the PCR instrument, then removed and incubated at room temperature for 3 min, treated in liquid nitrogen for 3 min, and circulated 3 times. Then the protein was detected and quantified by Western blotting analysis.

### Orthotopic pancreatic cancer mouse model

The establishment and treatment of orthotopic models were performed as reported previously [26]. 50 μl Panc1-luc/SW1990-luc cell suspension was injected into the pancreas of male SCID mice. When tumor volume was confirmed, the mice were randomly divided into two groups: the control group only received controls while the DHPO treatment group was administered by intraperitoneal injection. For the Panc1 orthotopic model, DHPO injection was at a dose of 5 mg/kg/day, 7 days/week, for 5 weeks. For the SW1990 orthotopic model, DHPO injection was at a dose of 10 mg/kg/day, 7 days/week, for 4 weeks. The tumor volume was monitored once every 7 days by fluorescence imaging via an IVIS imaging system. The body weight was measured every three days as a surrogate marker for toxicity. At the end of the treatment, the mice were sacrificed and their tumors and major organs were excised for subsequent experiments. Hematoxylin and eosin staining and scoring were performed as described previously [27].

### Statistical analysis

The data were analyzed using the Prism software program (version 9) (Graph Pad Software Inc., San Diego, CA, USA). Data were expressed as mean ± SEM. Statistical analyses between control and treatment groups were performed by student’s *t*-test. *P* < 0.05 was considered to be statistically significant.

## Results

### UbcH5c overexpression correlates with IAP expression and poor prognosis of pancreatic cancer patients

**Fig. 1:**
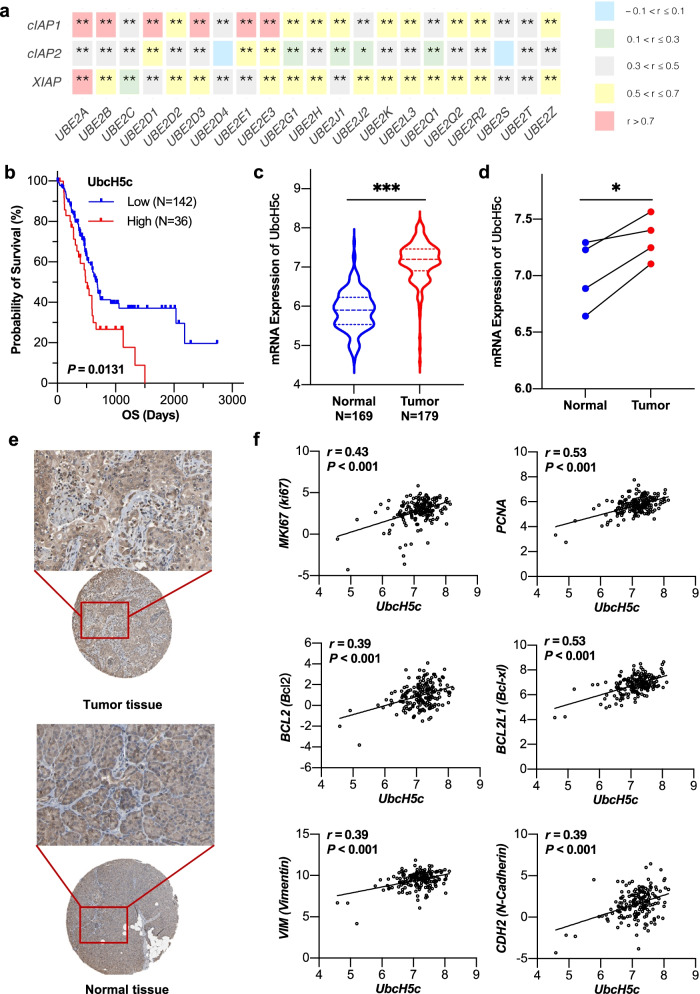
UbcH5c (also named UBE2D3) is overexpressed in pancreatic cancer and correlated with the poor prognosis of patients with this disease. **a** Heatmap showing mRNA expression level correlation of E2s with IAP family members, including *cIAP1/BIRC2*, *cIAP2/BIRC3*, and *XIAP*. **b** Kaplan–Meier survival analysis for the association of UbcH5c expression with overall survival of patients in the TCGA pancreatic cancer dataset. **c** *UbcH5c* mRNA expression is upregulated in the pancreatic tumor tissues compared with it in the normal tissues group from TCGA TARGET GTEx dataset. **d** *UbcH5c* mRNA expression is upregulated in the pancreatic tumor samples compared with paired adjacent nontumorous samples from TCGA dataset. **e** Representative IHC images on tissue microarray probed with the anti-UbcH5c antibody in Human Protein Atlas. **f** Correlation analysis of *UbcH5c* and indicated genes mRNA expression levels in the TCGA pancreatic cancer dataset. Correlation coefficient *r* and *P* values are shown on the graphs. * *P* < 0.05, ** *P* < 0.01, *** *P* < 0.001

To identify which E2 collaborates with IAPs for ubiquitination, we first compared the mRNA expression levels of 21 E2s between pancreatic tumor tissues and normal tissues and found that the mRNA expression levels of all these E2s were upregulated in tumor samples compared with them in normal tissues from TCGA TARGET GTEx datasets obtained from UCSX xena (Additional file 2: Fig. S1). In light of the overexpression of E2s in pancreatic cancer patients, we performed the correlation analysis of the IAP family and E2s at mRNA expression levels and identified six E2s as candidate drug targets. As shown in Fig. 1a, the mRNA expression levels of *UBE2A*, *UBE2B*, *UBE2D1*, *UBE2D3* (also known as *UbcH5c*), *UBE2E1*, and *UBE2E3* have a stronger positive correlation with *cIAP1*, and *UBE2A* was more correlated with *XIAP* (r > 0.7). Among six E2s, only *UbcH5c* had a negative impact on the overall survival time of patients with pancreatic cancer (Fig. 1b). Additionally, the mRNA expression of *UbcH5c* was significantly upregulated in tumor tissues relative to levels in normal tissues from TCGA TARGET GTEx datasets (Fig. 1c), and in the paired adjacent non-tumor tissues from TCGA dataset (Fig. 1d) using the online tool CVCDAP [28]. Also, the UbcH5c protein level was higher in the pancreatic tumor tissues than the normal tissues from the Human Protein Atlas (Fig. 1e). To further investigate the role of UbcH5c in pancreatic carcinogenesis, we employed bioinformatics to analyze the gene expression profile of pancreatic cancer patients from TCGA dataset. The correlation scatter plots demonstrated that *UbcH5c* expression was positively correlated with the expression of cell proliferation-related genes like ki-67 and PCNA, anti-apoptosis-related genes like Bcl2 and Bcl-xl, as well as EMT-related genes like Vimentin and N-Cadherin (Fig. 1f). Notably, recent studies have demonstrated that UbcH5c engages in the ubiquitination of dozens of proteins and modulates various pathways involved in cell proliferation, anti-apoptosis, and metastasis [29].

### Identification of a new small molecule UbcH5c inhibitor

**Fig. 2:**
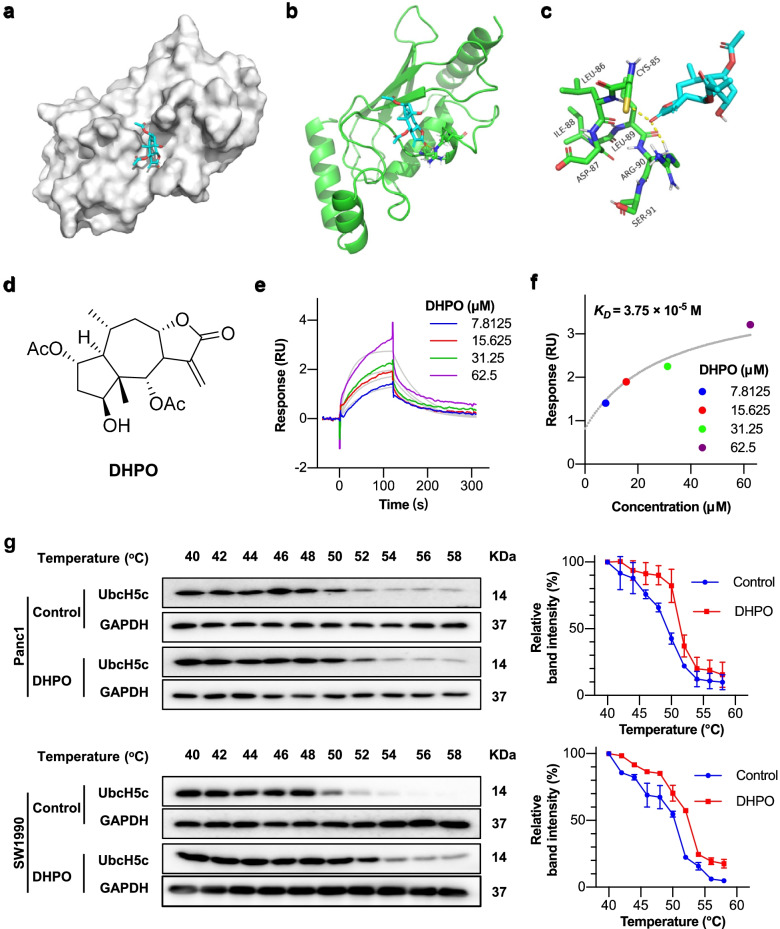
DHPO directly targets UbcH5c. **a**, **b** and **c** The predicted binding mode of DHPO to UbcH5c by docking analysis. **d** Chemical structure of DHPO. **e** and **f** The binding affinity of DHPO to UbcH5c was evaluated by surface plasmon resonance analysis. **g** The DHPO-UbcH5c binding was examined in Panc1 and SW1990 cells using cellular thermal shift assays and western blot

To identify small molecule inhibitors for UbcH5c, we performed a molecular docking and structure-based virtual screening assay against an in-house library of natural products [30, 31] using UbcH5c as a substrate, and an active compound termed 2α,6α-diacetoxy-4β-hydroxy-11(13)-pseudoguaien-12,8α-olide (DHPO) was discovered (Fig. 2d). The molecular docking results indicated that DHPO targeted UbcH5c by forming hydrogen bonds with the amino groups of Leu86 and Arg90 (Fig. 2a, b, and c). To further validate the direct interaction of DHPO with UbcH5c, their binding affinity was estimated by surface plasmon resonance assays. As shown in Fig. 2e and f, we observed that DHPO interacted with UbcH5c protein with an equilibrium dissociation constant (*K*_D_) of 3.75 × 10^–5^ M. The DHPO-UbcH5c binding was further confirmed by cellular thermal shift assays in Panc1 and SW1990 cells. The western blotting analysis showed the presence of UbcH5c at the lower test temperatures followed by its disappearance with the increased temperature in the control groups. However, the addition of DHPO resulted in a strong band of UbcH5c at 50 °C and the detectable protein bands even the temperature increased to 58 °C (Fig. 2g), which suggested that DHPO was effectively engaged by the UbcH5c protein in both Panc1 and SW1990 cells.

### DHPO exerts anticancer activities in pancreatic cancer cells in vitro

**Fig. 3:**
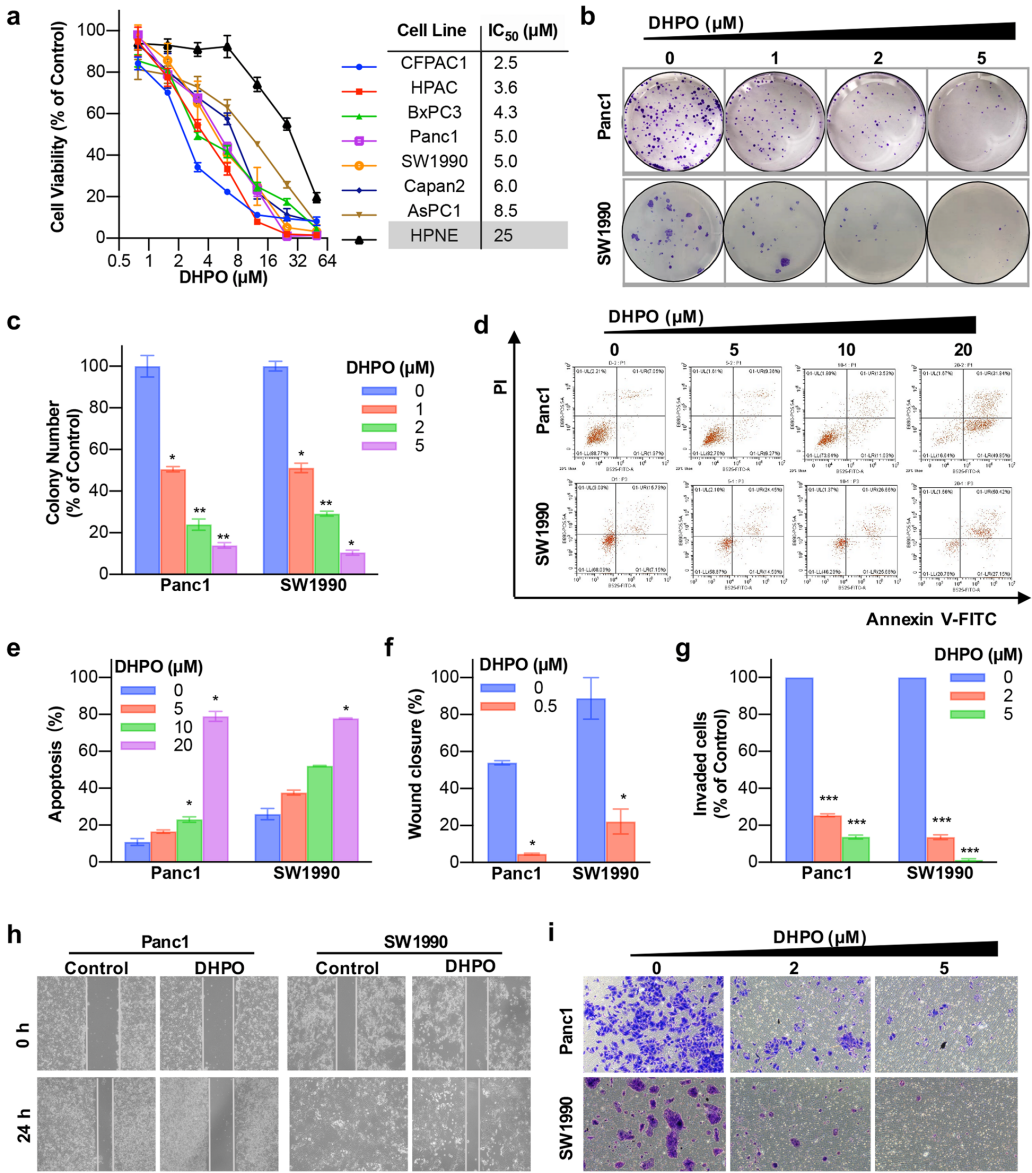
DHPO inhibits cell proliferation, triggers apoptosis, and prevents migration and invasion in pancreatic cancer cells in vitro. **a** Effects of DHPO on the viability of human pancreatic cancer cell lines versus normal pancreatic cells were evaluated by 72-h CCK8 assays. **b** and **c** The colony-formation assays were performed and colony numbers were counted. **d** and **e** Representative results of Annexin V-FITC/PI staining and quantitative analysis after the DHPO treatment for 48 h. **f** and **h** Wound healing assays after DHPO treatment for 24 h. **g** and **i** Transwell invasion assay was performed by the 24-transwell system, followed by quantitative analysis. * *P* < 0.05, ** *P* < 0.01, *** *P* < 0.001

To examine the inhibitory effects of DHPO on pancreatic cancer cell viability, seven pancreatic cancer cell lines, including CFPAC1, HPAC, BxPC3, Panc1, SW1990, Capan2, and AsPC1, and one normal pancreatic cell line HPNE were treated with different concentrations of DHPO for 72 h. As shown in Fig. 3a, DHPO significantly inhibited the growth of pancreatic cancer cell lines in a concentration-dependent manner, and its cytotoxicity was much greater in pancreatic cancer cell lines (IC_50_ = 2.5 ~ 8.5 μM) than that in the normal pancreatic cell line HPNE (IC_50_ = 25 μM). The colony formation assay showed that DHPO markedly declined the colony formation efficiency (Fig. 3b and c). To determine the effects of DHPO on cell apoptosis, an Annexin V–FITC dual staining assay was performed by flow cytometry. The results showed that DHPO induced pancreatic cancer cell apoptosis in a concentration-dependent way (Fig. 3d and e). We further performed wound healing assays and transwell invasion assays to assess the effects of DHPO on pancreatic cancer cell migration and invasion, respectively. As shown in Fig. 3f and h, DHPO significantly prevented the migration of both Panc1 and SW1990 cells. Similarly, DHPO concentration-dependently inhibited the invasion of both Panc1 and SW1990 cells (Fig. 3g and i).

### Transcriptome analysis revealed the involvement of the TNF-α/NF-κB pathway in anticancer activity of DHPO

**Fig. 4:**
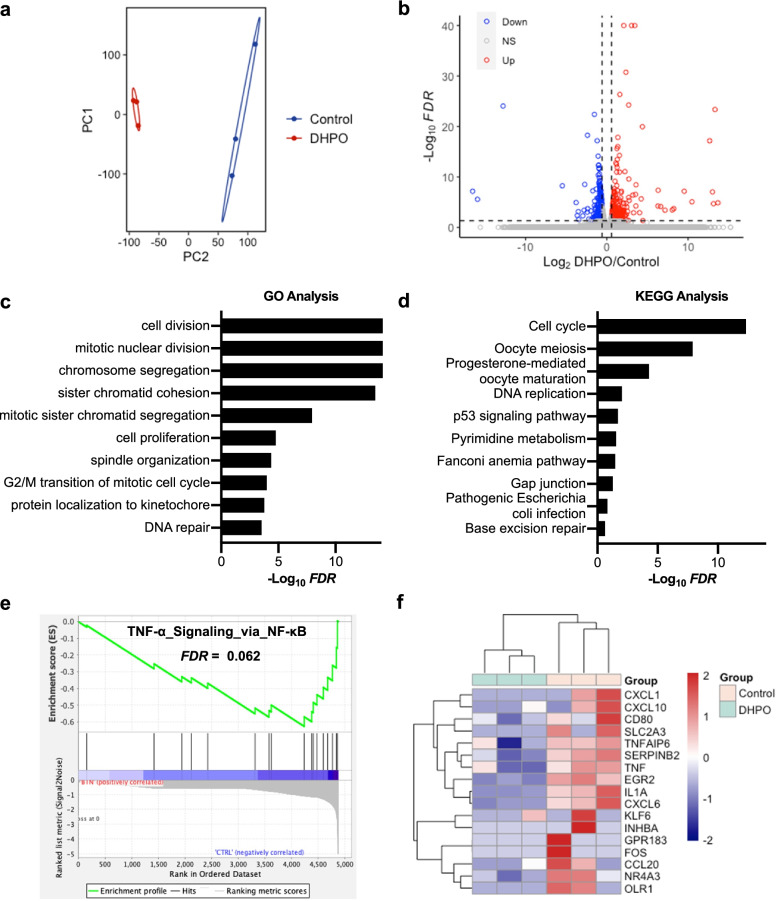
Transcriptome in Panc1 cells identifies downregulated pathways. **a** PCA of transcriptomes with or without DHPO treatment represented in a two-dimensional space. **b** Volcano plot showed the differentially expressed genes between DHPO-treated and untreated Panc1 cells. **c** and **d** GO analysis and KEGG analysis for the significantly downregulated genes by DHPO compared to control in Panc1 cells. The top 10 downregulated pathways were listed. **e** GSEA results demonstrating a strong association with TNFα signaling via NF-κB (from hallmark MSigDB signatures), following DHPO treatment in Panc1 cells. **f** Heatmap shows expression levels of TNFα signaling via NF-κB genes in Panc1 cells with or without DHPO treatment

To investigate the molecular mechanisms of how DHPO inhibits proliferation and migration in pancreatic cancer cells, we performed RNA sequencing of Panc1 cells after DHPO treatment using DMSO treatment as control. Indeed, principal-component analysis (PCA) showed a shift in principal component 1 (PC1) and PC2 in DHPO versus control (Fig. 4a). After the preliminary screening, a total of 527 genes were identified as differentially expressed genes. Of these, 323 genes were upregulated and 204 genes were downregulated in the DHPO group (Fig. 4b). To gain insight into the global patterns of the effects of downregulated genes on the biological processes, we performed the GO and KEGG analysis. Briefly, the results revealed that downregulated genes after DHPO treatment were mainly involved in biological processes or signaling pathways including cell cycle, cell division, cell proliferation, DNA repair, and DNA replication (Fig. 4c and d), which was further confirmed that DHPO inhibited proliferation and induced apoptosis of Panc1 cells. To gain a more comprehensive understanding of the pathways altered in response to DHPO versus control, we performed Gene Set Enrichment Analysis (GSEA) [32] on downregulated genes. For the Hallmark gene sets, enrichment results revealed that TNF-α signaling via NF-κB was enriched in the control group (Fig. 4e and Additional file 3: Table S2). This finding suggested that DHPO may exert anticancer activities by repressing the TNF-α/NF-κB signaling. A heatmap of genes in the TNF-α signaling via NF-κB gene set was generated from transcriptome data (Fig. 4f). It was speculated that direct interaction of DHPO with UbcH5c decreased the ubiquitination and degradation of IκBα and inhibited NF-κB activation as well as the expression of its downstream target genes [29].

### Proteomic analysis further confirmed the anticancer activity of DHPO

**Fig. 5:**
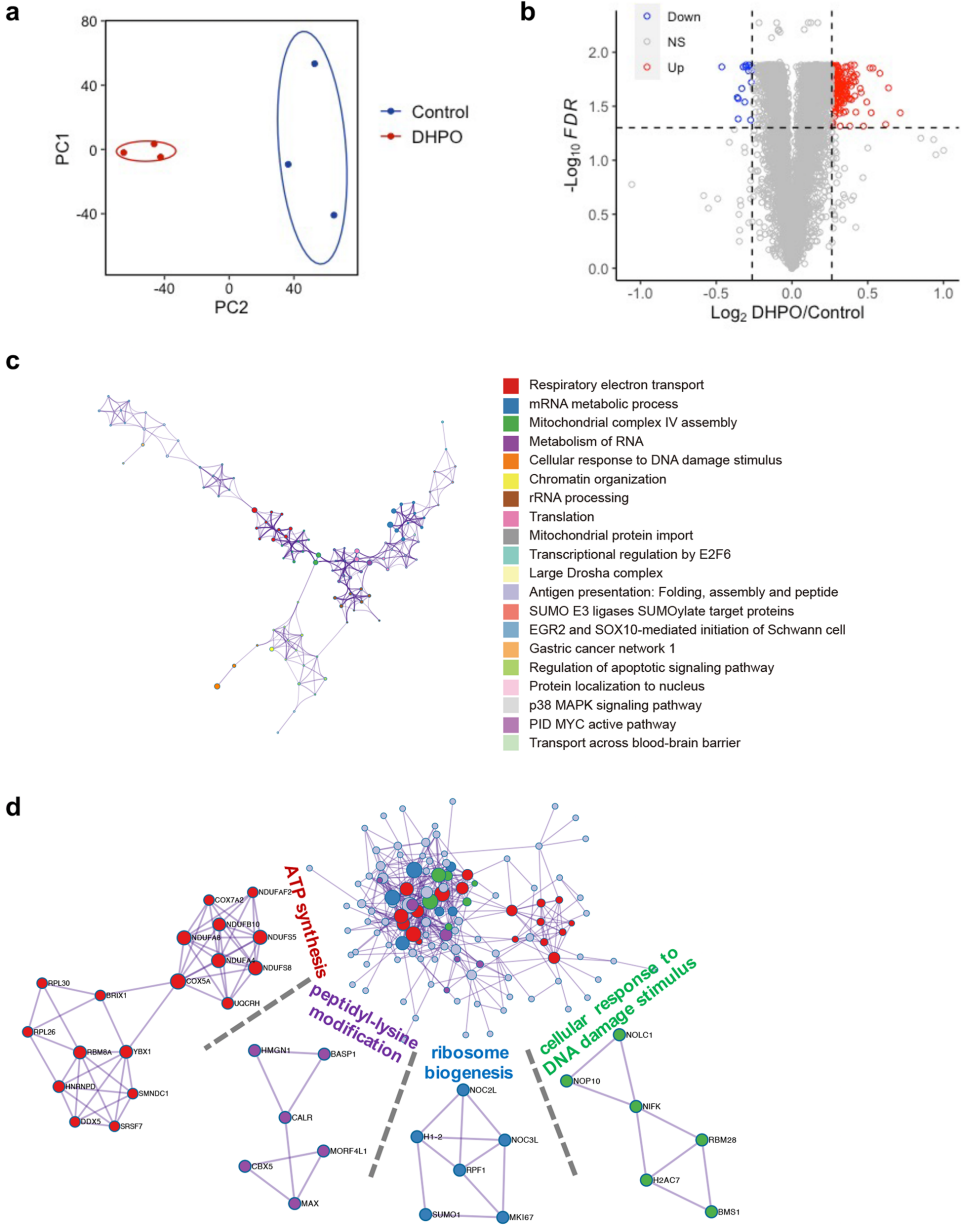
Proteomics in Panc1 cells shows enriched pathways. **a** PCA of proteomics with or without DHPO treatment represented in a two-dimensional space. **b** Volcano plot showed the differentially expressed proteins between DHPO-treated and untreated Panc1 cells. **c** Network shows pathway enrichment. Each term is identified by color. Size of clusters is proportional to the number of input proteins included in the term. **d** Protein–protein interaction analysis of input proteins. The center node represents the full interactome, and the surrounding nodes are identified according to the representative functions included. Each node is colored according to the function

To further elucidate the underlying mechanisms of DHPO exhibiting anticancer performance, we conducted the multiplexed tandem mass tag (TMT) quantitative proteomics of Panc1 cells treated with or without DHPO. A total of 5505 unique peptides were identified and annotated in all samples. PCA showed a shift in PC1 and PC2 in DHPO versus control (Fig. 5a). As shown in Fig. 5b, differentially expressed proteins between DHPO-treated and untreated Panc1 cells were determined. Compared with the control group, the expression levels of 163 proteins were significantly altered following DHPO treatment. Among them, 15 proteins were downregulated and 148 proteins were upregulated. To comprehensively understand the functions of the differentially expressed proteins, we performed a pathway and protein–protein interaction enrichment and clustering analysis with Metascape [33]. Analysis of pathway enrichment revealed pathways related to the regulation of apoptotic signaling pathway, protein localization to nucleus, MYC active pathway, antigen presentation, and RNA metabolism (Fig. 5c), suggesting the general effects of DHPO on cell proliferation and cell death. Next, we used the molecular complex detection algorithm to identify protein–protein interaction clusters involved pathways, including metabolism and cellular response to DNA damage stimulus (Fig. 5d).

### DHPO inhibits TNF-α-induced activation of the NF-κB pathway

**Fig. 6:**
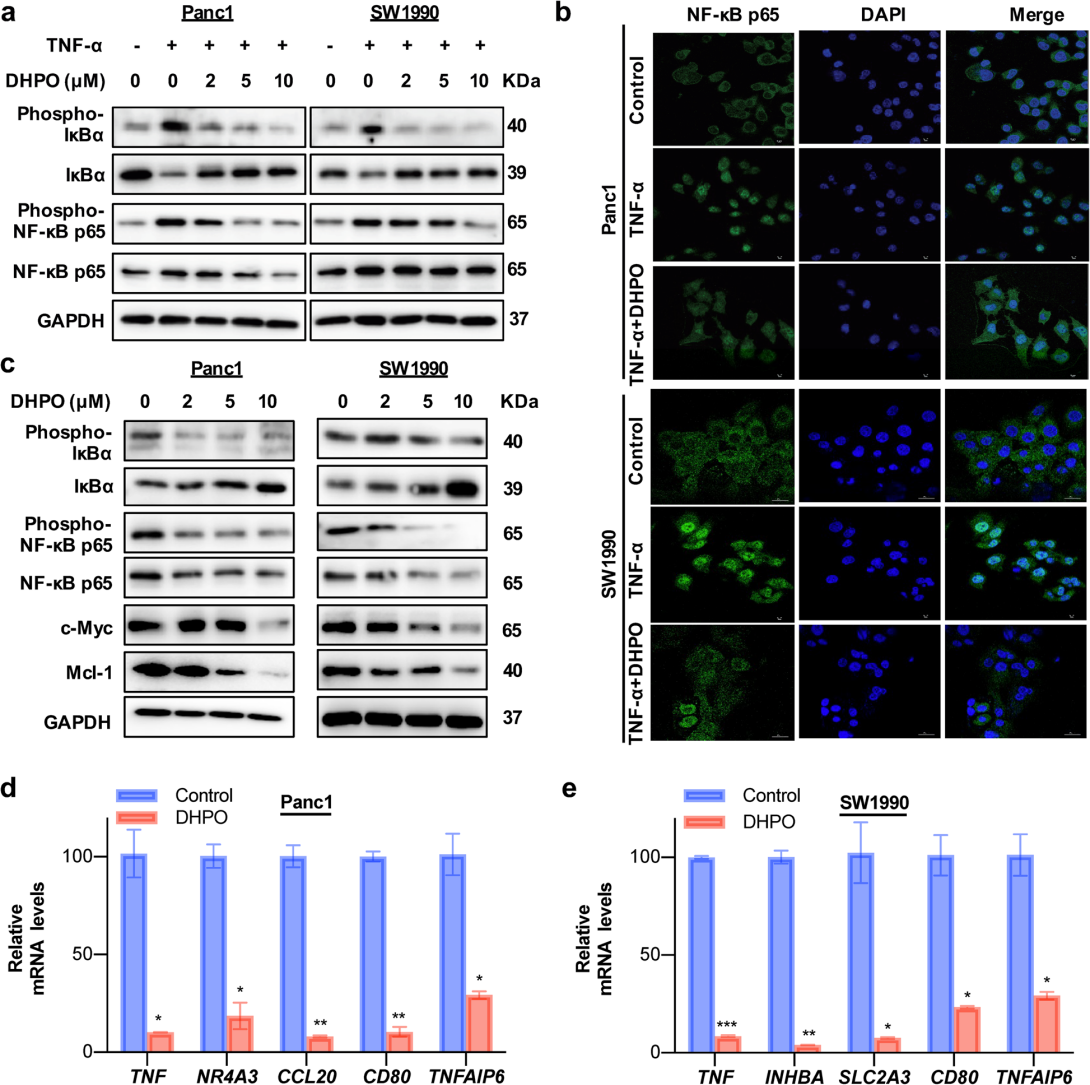
DHPO inhibits pancreatic cancer cell growth and metastasis by inhibiting the NF-κB pathway. **a** The protein expression levels of IκBα, phosphorylated IκBα, NF-κB p65, and phosphorylated NF-κB p65 were detected by western blotting analysis after treatment with DHPO for 24 h and stimulation with TNF-α (20 nM) for another 15 min. **b** The effects of DHPO on the expression and distribution of NF-κB p65 were explored by immunofluorescence in the indicated cells with or without TNF-α (20 nM) stimulation. **c** The protein expression levels of IκBα, phosphorylated IκBα, NF-κB p65, phosphorylated NF-κB p65, and downstream target gene products (c-Myc and Mcl-1) of NF-κB pathway were detected by western blotting analysis after DHPO treatment for 24 h. **d** and **e** The relative mRNA levels of indicated genes in Panc1 and SW1990 cells were determined by real-time qRT-PCR. * *P* < 0.05, ** *P* < 0.01, *** *P* < 0.001

We then evaluated the effects of DHPO on TNF-α-induced activation of the NF-κB pathway. As shown in Fig. 6a, DHPO concentration-dependently inhibited the phosphorylation of IκBα, stabilized IκBα, and decreased the levels of total and phosphorylated NF-κB p65 in both Panc1 and SW1990 cells upon TNF-α treatment. We also found that DHPO significantly reduced the nuclear translocation of NF-κB p65 after TNF-α stimulation (Fig. 6b). To gain insights into the consequence of the decreased NF-κB-driven transcriptional activity in DHPO-treated Panc1 and SW1990 cells, the western blotting analysis was performed and showed that DHPO treatment markedly downregulated the expression of the downstream genes, c-Myc (drivers of the cell cycle) and Mcl-1 (anti-apoptosis) (Fig. 6c). We also used real-time qRT-PCR to confirm the expression of TNF-α/NF-κB signaling-related genes in both Panc1 and SW1990 cells treated with DHPO or DMSO. DHPO treatment caused significant repression of all the examined TNF-α/NF-κB signaling-related genes compared to the control (Fig. 6d and e).

### UbcH5c plays a critical role in DHPO’s anticancer activity

**Fig. 7:**
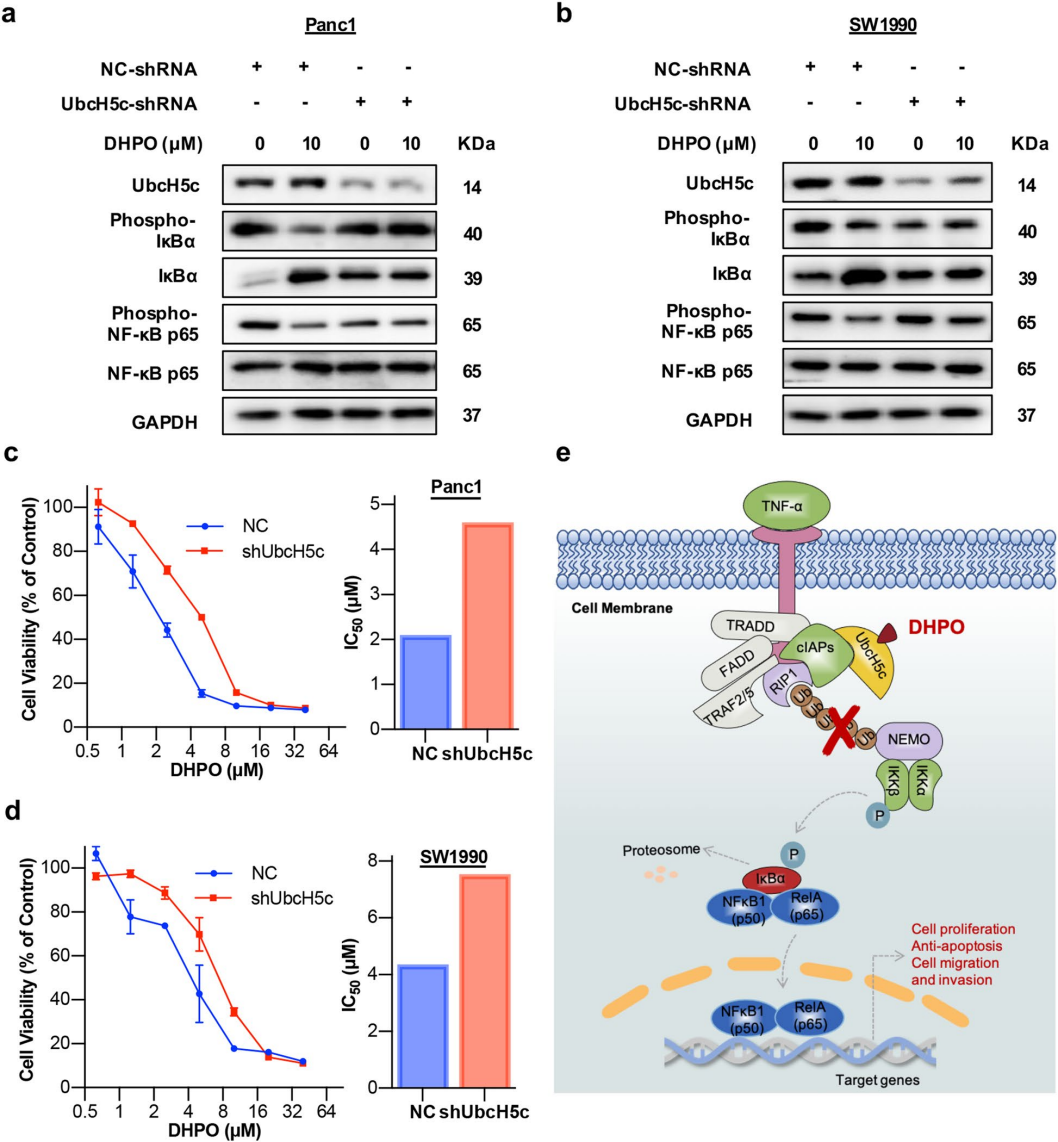
UbcH5c knockdown decreases the anticancer activity of DHPO. **a** and **b** The expression levels of indicated proteins in Panc1 and SW1990 cells transfected with shRNA targeting UbcH5c or control shRNA were determined by western blotting analysis after treatment with DHPO for 24 h. **c** and **d** The 72-h cell viability assays in indicated cell lines transfected with shRNA targeting UbcH5c or control shRNA. **e** Scheme showing a proposed molecular mechanism for the anticancer activity of DHPO

To further examine whether the binding of DHPO to UbcH5c results in anticancer activity, we generated lentiviral short hairpin RNA (lenti-shRNA) vectors to knockdown (KD) UbcH5c in the Panc1 and SW1990 cells. UbcH5c expression was significantly silenced by UbcH5c shRNA relative to the control shRNA (Fig. 7a and b). The western blotting assay revealed that UbcH5c KD specifically blocked DHPO-induced IκBα dephosphorylation and stabilization and thereby reversed the inhibitory effects of DHPO on NF-κB p65 phosphorylation and activation in Panc1 and SW1990 cells (Fig. 7a and b). The cell viability assay showed that UbcH5c KD reduced the inhibitory effects of DHPO on cell viability compared with the control cells (Fig. 7c and d).

### DHPO suppresses orthotopic pancreatic tumor growth and metastasis in vivo, without causing significant host toxicity

**Fig. 8:**
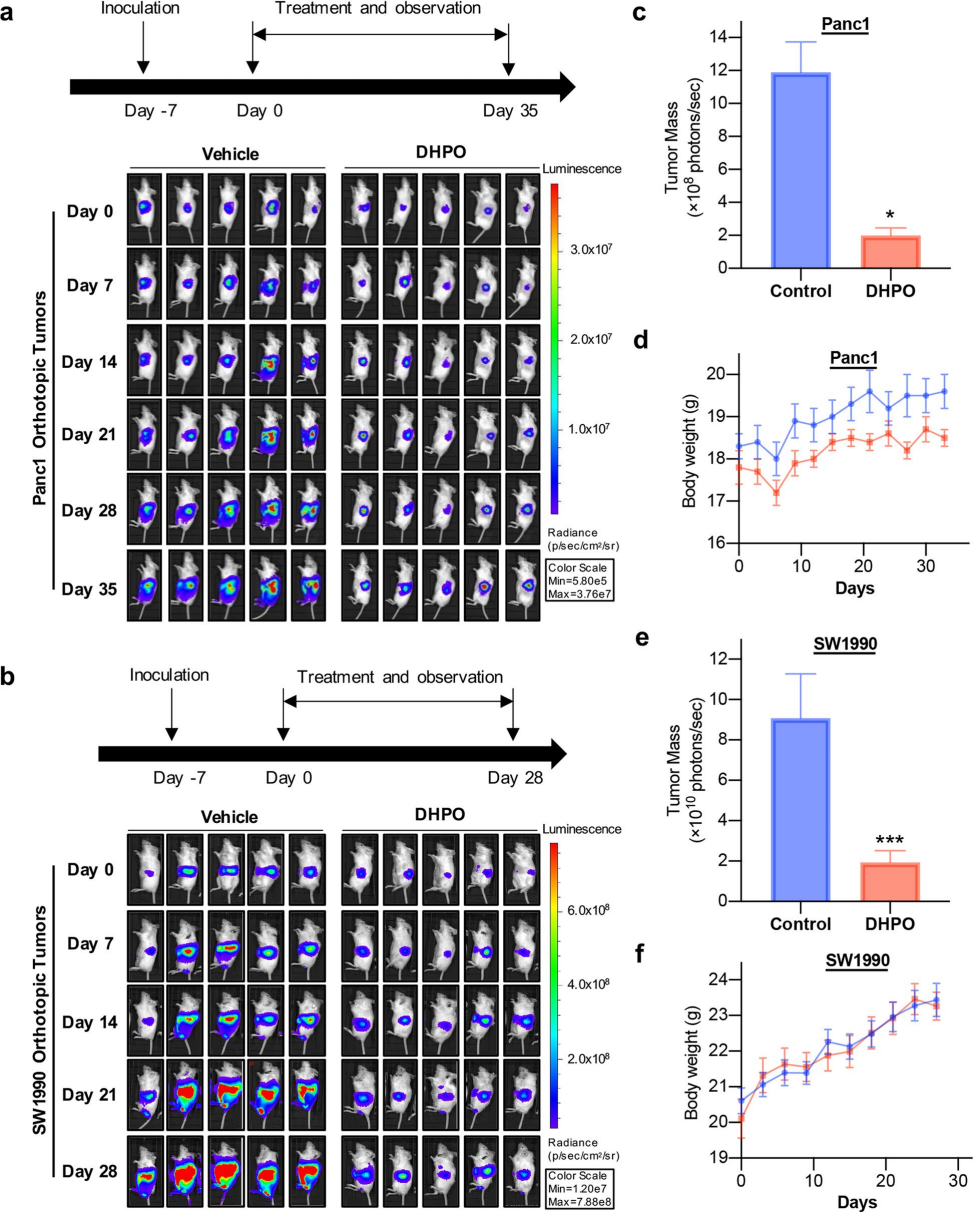
DHPO suppresses orthotopic pancreatic tumor growth in vivo. **a** and **b** Representative bioluminescent images for Panc1-luc and SW1990-luc orthotopic pancreatic tumors at different time points post treatment. **c** and **e** At the end of the experiment, the average tumor mass of the DHPO-treated mice was compared with that of the control mice. **d** and **f** Changes in body weights of mice treated with DHPO or vehicle at different treatment periods. * *P* < 0.05, *** *P* < 0.001

To study the anticancer efficacy of DHPO in vivo, Panc1 and SW1990 orthotopic tumor models were established and treated with control or DHPO by intraperitoneal injection for 35 and 28 days, respectively, then bioluminescence signals were directly monitored by an in vivo imaging system. The results showed that the control group exhibited rapid tumor growth, whereas the group receiving DHPO treatment notably suppressed tumor growth (Fig. 8a and b). As shown in Fig. 8c and e, compared to the control mice, DHPO inhibited tumor growth by 80.67% and 78.74% in Panc1 and SW1990 orthotopic tumor models, respectively. To monitor the potential in vivo side effects of DHPO, body weight was measured every three days. There was no significant difference in terms of average body weights (Fig. 8d and f), which suggested that DHPO did not cause any notable host toxicity at the effective dose.

**Fig. 9:**
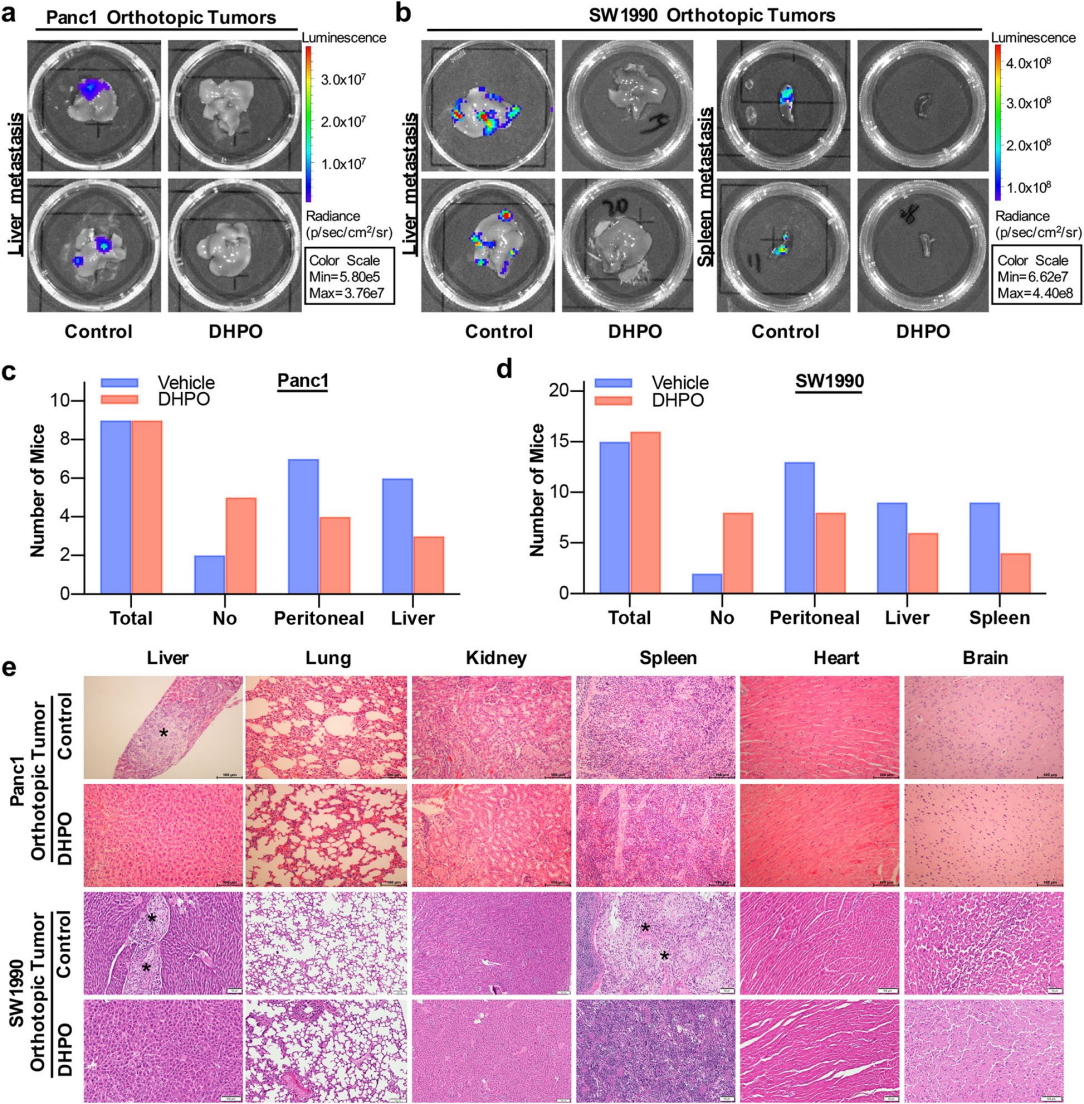
DHPO prevents metastasis of pancreatic cancer in vivo. **a** and **b** Representative images of liver and spleen metastasis in Panc1 and SW1990 orthotopic models. **c** and **d** The number of mice with metastasis to the peritoneum, liver, and spleen in Panc1 and SW1990 orthotopic models. **e** Representative hematoxylin and eosin staining images of various organs. Scale bar = 5 μm. * represents tumor metastasis

We further assessed the effects of DHPO on pancreatic tumor metastasis in vivo. As indicated in Fig. 9a and b, DHPO treatment significantly prevented the metastasis of both Panc1 and SW1990 tumors to liver, peritoneum, and spleen. Compared with the control-treated mice, DHPO decreased the incidence of peritoneal and liver metastasis in mice bearing orthotopic Panc1 tumors from 7/9 and 6/9 to 4/9 and 3/9 mice, respectively (Fig. 9c), and decreased the incidence of peritoneal, liver, and spleen metastasis in mice bearing orthotopic SW1990 tumors from 13/15, 9/15 and 9/15 to 8/16, 6/16 and 4/16 mice, respectively (Fig. 9d). The vital organs, including liver, lungs, kidneys, spleen, heart, and brain were also harvested from mice for histologic examinations. No histological changes in these organs were found in the DHPO treatment groups whereas there were liver and spleen metastasis in control groups (Fig. 9e), which confirmed that DHPO has no significant host toxicity at the effective doses.

## Discussion

New therapeutic targets are critically needed for the development of promising anti-pancreatic cancer therapeutic drugs [34–36]. We have recently proposed that UbcH5c is a potential therapeutic target in human cancer [29]. Indeed, there is increasing evidence that UbcH5c is overexpressed in human cancer, such as esophageal squamous carcinoma and breast cancer [37–39]. UbcH5c was also found to be upregulated in plasma samples of colorectal cancer, and the overexpression of UbcH5c promotes metastasis of colorectal cancer. Here we found that UbcH5c is overexpressed in pancreatic tumor tissues compared with non-tumor tissues and predicts a poor prognosis in patients with pancreatic cancer. Our experimental data indicated that UbcH5c may serve as a promising biomarker and therapeutic target for the treatment of pancreatic cancer patients. Further characterization and verification of UbcH5c need to be carried out in more clinical samples from multicenter cohorts and some important clinicopathologic features should also be considered.

It is worth noting that selective inhibition of UbcH5c remains an active area of drug development. To date, several UbcH5c inhibitors have been developed to inactivate UbcH5c by forming a covalent adduct in recent pharmacodynamics studies [40], and they have been reported to exert anti-inflammatory activity and increase drug sensitivity in various models [30, 39, 41]. Here, we identified a novel UbcH5c inhibitor, DHPO, a sesquiterpene lactone isolated from *Inula* plants, which is widely distributed in China and has been used as a traditional Chinese medicine Jinfeicao for treating various diseases, including cancer [22, 42]. This compound has been reported to show potent Nrf2-activating and neutral protective activities [43], but its role in cancer is not clear. The present study indicated that DHPO could directly bind to UbcH5c, potently inhibit pancreatic cancer cell proliferation, induce apoptosis, and prevent migration and invasion in vitro. Moreover, DHPO suppressed pancreatic tumor growth and metastasis in vivo, without causing any obvious host toxicity. Our work represents the first attempt to demonstrate that targeting UbcH5c could be an alternative strategy to control pancreatic cancer progression. However, more specific and effective UbcH5c inhibitors with higher binding affinity are expected to be identified via virtual screening of other compound libraries and validation of cell-based assays. The structural optimization of DHPO is also a possible strategy to develop more specific UbcH5c inhibitors. Moreover, DHPO may also exert anticancer efficacy in other types of cancer with high expression levels of UbcH5c. The efficacy and safety of DHPO and other UbcH5c inhibitors should be evaluated in more clinically relevant cancer models in the future.

Recent studies have reported that UbcH5c acts as an oncoprotein by inducing the ubiquitination and degradation of dozens of proteins and thereby causing NF-κB activation [29]. It is becoming clear that NF-κB has multiple roles in cell proliferation, survival, migration, tumorigenesis, and metastasis in cancer; inhibition of this pathway could be a therapeutic option [44]. Over the past few years, a great number of small molecules mainly targeting proteasome [45] or IKK [46] have been developed to block NF-κB activation and some of them are being investigated in clinical trials. However, limited or no efficacy of these small molecules has been observed in pancreatic cancers [47], leading us to identify new targets upon which the development of new agents inhibiting NF-κB will be based. Based on our results, we proposed the molecular mechanism of DHPO against pancreatic cancer (Fig. 7e). This compound may directly bind to UbcH5c and disrupt the E2/E3 interaction of the UbcH5c/cIAPs complex, thus inhibiting the polyubiquitination of receptor-interacting protein 1 (RIP1) and NF-κB essential modulator (NEMO). Consequently, the IKK-β-mediated phosphorylation of IκBα and NF-κB is blocked by DHPO, thereby repressing the transcription of downstream target genes in pancreatic cancer cells. Further, we demonstrated that UbcH5c KD cells were resistant to DHPO treatment, showing less sensitivity in inhibiting cell viability. Nonetheless, these results must be interpreted with caution and a number of limitations should be borne in mind. First, the specificity of DHPO for UbcH5c over other E2s is not clear. Second, DHPO affects ubiquitination of which members of cIAPs should be validated using cutting-edge methods. Third, UbcH5c was also involved in many cancer-related signaling pathways, including the p53 signaling pathway [39, 48], p62-mediated autophagy [49], YAP pathway [50], and DNA repair pathways [37], so whether DHPO also inhibits pancreatic cancer via these pathways needs to be further explored. In addition, DHPO may have secondary molecular targets, which should also be studied in the future.

## Conclusions

In the present study, we demonstrated the clinical relevance of UbcH5c with pancreatic cancer and identified a potent UbcH5c inhibitor DHPO, which showed anticancer efficacy in pancreatic cancer cell models in vitro and in vivo via inhibiting the NF-κB signaling pathway. In summary, our results provide a novel and promising target for pancreatic cancer and a drug candidate for the treatment of human cancer.

## Supplementary Information

**Additional file 1:** **Table S1.** Primers used in the study.

**Additional file 2:** **Figure S1.** Landscape of E2s between pancreatic cancer tissues and normal tissues.

**Additional file 3:** **Table S2.** GSEA results.

## Data Availability

The datasets obtained and analyzed during the current study were made available from the corresponding authors through request.

## Abbreviations

HECT: Homologous to E6AP C-terminus
RING: Really interesting new gene
RBR: RING-between-RING
IAPs: Inhibitor of apoptosis proteins
EMT: Epithelial-mesenchymal transition
PCA: Principal-component analysis
GSEA: Gene set enrichment analysis
TMT: Tandem mass tag
