# Supplementary material for: Targeting E2 ubiquitin-conjugating enzyme UbcH5c by small molecule inhibitor suppresses pancreatic cancer growth and metastasis
Source: Mol Cancer. 2022 Mar 10;21:70. doi: 10.1186/s12943-022-01538-4 (PMC8908661; doi:10.1186/s12943-022-01538-4)
Supplement: Supplementary file 1 — Additional file 1: Table S1. Primers used in the study. [file 12943_2022_1538_MOESM1_ESM.docx]

**Additional file 1:**

Table S1. Primers used in the study.

| Gene | Sequence |
| --- | --- |
| *TNF* | Forward: CCTCTCTCTAATCAGCCCTCTG |
|  | Reverse: GAGGACCTGGGAGTAGATGAG |
| *NR4A3* | Forward: ATAGTCTGAAAGGGAGGAGAGGTC |
|  | Reverse: TCTGGGTGTTGAGTCTGTTAAAGC |
| *CCL20* | Forward: TGCTGTACCAAGAGTTTGCTC |
|  | Reverse: CGCACACAGACAACTTTTTCTTT |
| *CD80* | Forward: AAACTCGCATCTACTGGCAAA |
|  | Reverse: GGTTCTTGTACTCGGGCCATA |
| *TNFAIP6* | Forward: TTTCTCTTGCTATGGGAAGACAC |
|  | Reverse: GAGCTTGTATTTGCCAGACCG |
| *INHBA* | Forward: CCTCCCAAAGGATGTACCCAA |
|  | Reverse: CTCTATCTCCACATACCCGTTCT |
| *SLC2A3* | GCTGGGCATCGTTGTTGGA |
|  | Reverse: GCACTTTGTAGGATAGCAGGAAG |
