# Supplementary figures and images for: Targeting E2 ubiquitin-conjugating enzyme UbcH5c by small molecule inhibitor suppresses pancreatic cancer growth and metastasis
Source: Mol Cancer. 2022 Mar 10;21:70. doi: 10.1186/s12943-022-01538-4 (PMC8908661; doi:10.1186/s12943-022-01538-4)

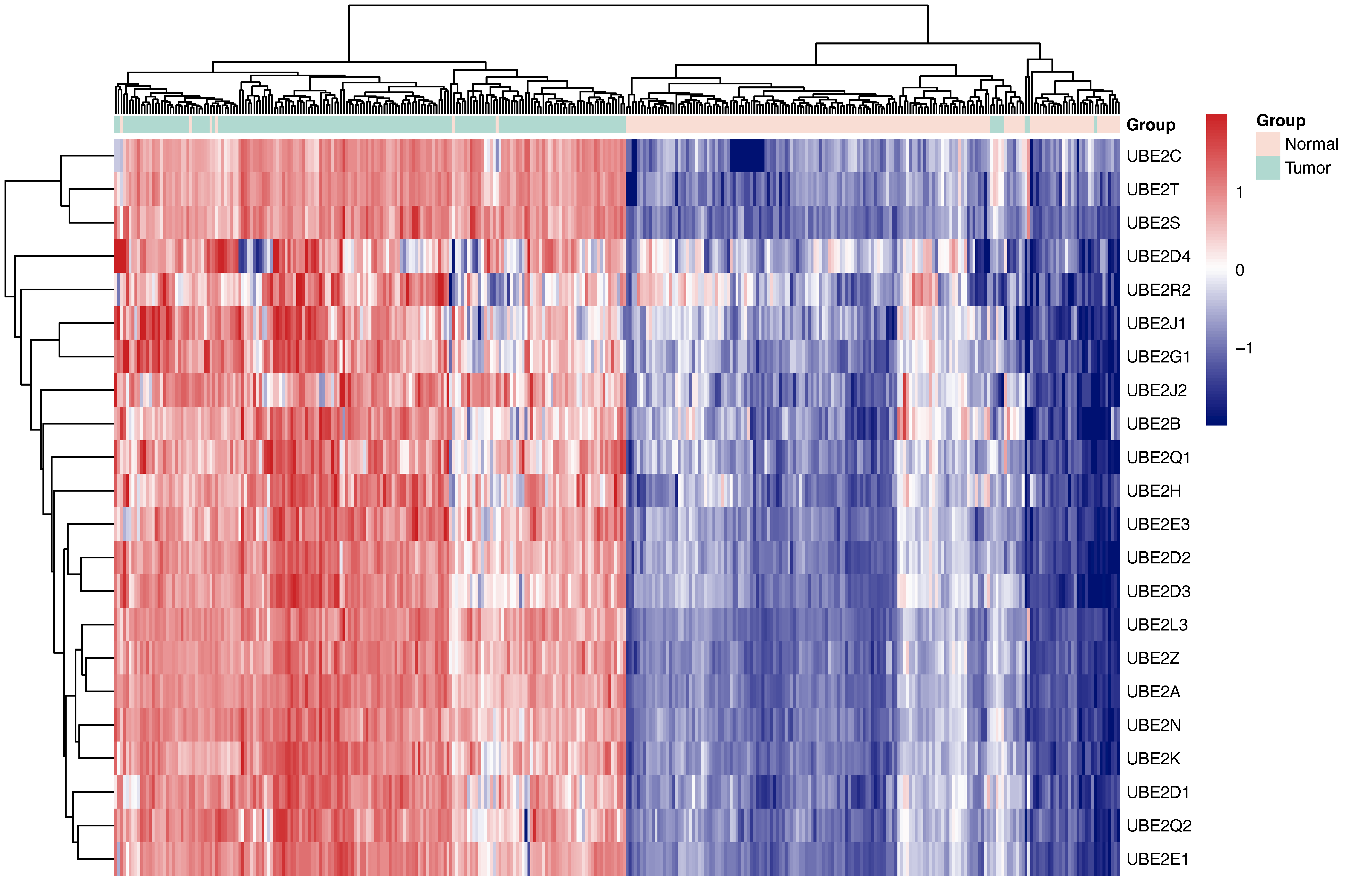

Supplement: Supplementary file 2 — Additional file 2: Figure S1. Landscape of E2s between pancreatic cancer tissues and normal tissues. [file 12943_2022_1538_MOESM2_ESM.tiff]
