# Supplementary material for: Targeting E2 ubiquitin-conjugating enzyme UbcH5c by small molecule inhibitor suppresses pancreatic cancer growth and metastasis
Source: Mol Cancer. 2022 Mar 10;21:70. doi: 10.1186/s12943-022-01538-4 (PMC8908661; doi:10.1186/s12943-022-01538-4)
Supplement: Supplementary file 3 — Additional file 3: Table S2. GSEA results. [file 12943_2022_1538_MOESM3_ESM.docx]

**Additional file 3:**

Table S2. GSEA results.

| Name | Size | NES | FDR |
| --- | --- | --- | --- |
| Spermatogenesis | 16 | -2.299 | 0.062 |
| TNF-α signaling via NFκB | 17 | -1.767 | 0.062 |
| E2F targets | 33 | -1.766 | 0.062 |
| G2M checkpoint | 37 | -1.686 | 0.080 |
| Mitotic spindle | 26 | -1.683 | 0.077 |
| Epithelial mesenchymal transition | 16 | -1.646 | 0.074 |
| Interferon γ response | 19 | -1.603 | 0.072 |
| Myogenesis | 18 | -1.526 | 0.083 |
| Allograft rejection | 23 | -1.476 | 0.085 |
| Inflammatory response | 23 | -1.410 | 0.089 |
| KRAS signaling up | 17 | -1.311 | 0.133 |
| Apical junction | 18 | -1.281 | 0.147 |
| Complement | 16 | -1.206 | 0.164 |
